# Comparing volitional electromyographic control strategies: from proportional mapping to muscle modeling and reflex augmentation

**DOI:** 10.1007/s13534-026-00594-9

**Published:** 2026-06-20

**Authors:** Matthias Voß, Rodrigo J. Velasco-Guillen, Philipp Beckerle

**Affiliations:** 1 Chair of Autonomous Systems and Mechatronics, Friedrich-Alexander-Universität Erlangen-Nürnberg, Erlangen, Germany; 2 Department Artificial Intelligence in Biomedical Engineering, Friedrich-Alexander-Universität Erlangen-Nürnberg, Erlangen, Germany

**Keywords:** Volitional control, EMG, Lower-limb, Reflexes, Muscle models, Prosthesis control

## Abstract

To advance intuitive volitional control for lower-limb prostheses, this study compares three torque control architectures based on electromyography (EMG) – including a novel reflex-augmented approach – using a virtual balance task. The goal is to determine whether muscle-model-based control improves performance and reduces user load relative to proportional EMG control, and whether incorporating simulated stretch reflexes further enhances results. Sixteen participants performed a virtual balancing task using EMG signals from antagonist lower-leg muscles to control an inverted pendulum subjected to perturbations. Four controllers were tested in randomized order: proportional, model-based, and two reflex-augmented variants differing in reflex gain. The model-based controller employed paired Hill-type muscle models; reflex variants additionally injected velocity-dependent stretch reflex feedback. User load was captured alongside control performance. While all participants successfully used all controllers, the model-based controller consistently enabled more stable and less effortful control than proportional EMG control. Reflex augmentation altered pendulum dynamics and showed slight performance tendencies in favor of the weaker reflex gain but did not yield clear or consistent benefits across participants compared with the model-based controller alone. Overall, incorporating muscle mechanics into EMG-based control substantially improves intuitive torque regulation over simple proportional mapping. While reflex augmentation showed promising potential, its current formulation did not provide reliable added value, indicating a need for refined reflex modeling and parameter tuning for future volitional prosthesis control applications.

## Introduction

Research on active lower-limb prostheses has grown substantially over the past two decades. Unlike passive devices that only store and return energy, or semi-active systems that modulate this return, active prostheses can deliver net positive power to the user. Beyond energetic advantages, active devices have been shown to enhance balance  [1], functional performance, satisfaction, and quality of life compared to passive prostheses  [2]. A major challenge in their development lies in designing control strategies that truly benefit users during locomotion  [3]. Long-term acceptance and consistent use depend on user satisfaction, which is closely linked to prosthesis embodiment  [4]. Control architectures play a central role here: intuitive and versatile systems promote independence, confidence, and safety  [5], while limited volitional governance can lead to frustration  [6, 7].

This highlights the need for control methods that allow users to intentionally command prosthesis behavior across tasks and environments – an approach termed volitional control. Surface electromyography (EMG) offers a mature, noninvasive means to achieve this by translating residual muscle activity into control commands  [8–11]. However, EMG-based control faces challenges such as interindividual variability and coactivation of antagonistic muscles  [12]. Encouragingly, users can improve control proficiency with training  [13, 14].

Despite these advantages, most active lower-limb prostheses today rely on autonomous, state-based control  [15]. No commercial devices currently employ EMG control  [15, 16], and even in research, only about half of EMG-based prototypes can be controlled volitionally  [17]. The creation of volitional controllers that ensure user safety and generalize across individual users and tasks remains an open research problem  [18]. A variety of EMG control architectures exist for lower-limb prosthetics and assistive devices in general, but their performance is difficult to compare due to a lack of standardized procedures and measures  [17].

We previously motivated the development of universally applicable volitional control architectures for everyday prosthesis use  [19]. Besides basing the controller on muscle and joint models, one novel suggestion was to incorporate simulated muscle reflexes in EMG control, based on their apparent relevance in human gait  [20, 21]. While hybrid controllers combining model-based EMG control with non-volitional methods exist  [22], we are not aware of any lower-limb EMG control studies implementing reflex integration. However, a similar approach has been successfully employed in a prosthetic hand  [23, 24].

In this paper, this concept is tested in a virtual EMG control experiment. Without the need for prosthesis hardware, we examine an EMG-driven torque controller that approximates human muscle dynamics by employing Hill-type muscle models  [25]. Expanding this control approach, we present a reflex model to be incorporated in the muscle simulation. Furthermore, we test these model-based control variants alongside a simple proportional EMG controller, facilitating a direct performance comparison. Proportional control is the simplest method of incorporating EMG inputs, yet it is commonly used and is recognized in recent reviews [16, 18]. As the sole control input or in combination with autonomous control signals, it has been employed in a variety of devices [26–28]. It was also used in a very similar virtual control experiment with transtibial amputees [29] and is consequently included here.

The presented controllers are evaluated in a user study on the virtual control experiment to answer two research questions:

1. Does a model-based EMG controller improve control performance and user load over a proportional EMG controller?
2. Does the incorporation of simulated muscle reflex improve control performance and user load over the model-based approach without reflexes?

In a preliminary version of this work, the reflex augmentation indicated performance and user load improvements upon the model-based controller, which in turn outperformed proportional control  [30]. The present article extends that work by increasing the number of participants from three to 16, adding a second reflex intensity, and providing a statistical analysis and more comprehensive discussion.

The paper is organized as follows. Section 2 details the experimental setup, the methodology for simulation and torque calculations, and the experimental protocol for the user study. Section 3 presents the results of the study. Finally, discussion and conclusions are presented in Sects. 4 and 5.

## Methods

**Fig. 1:**
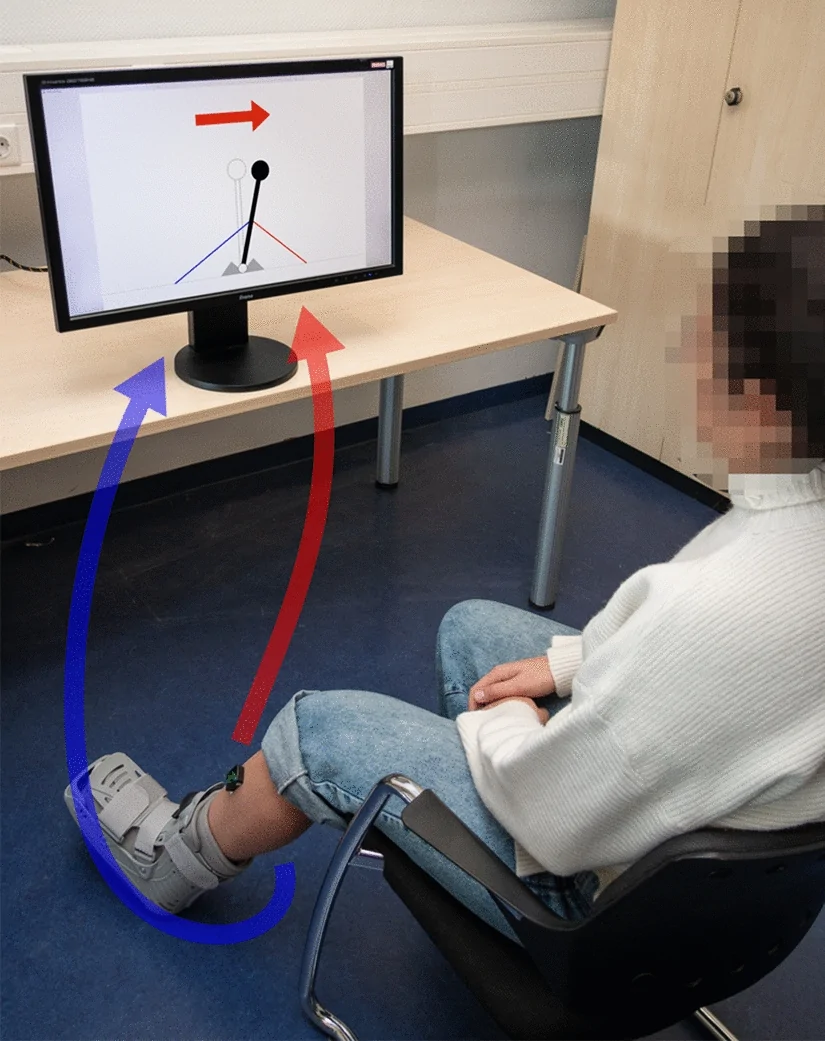
Photo of the experimental setup. A user controls the inverted pendulum via EMG signals from their lower leg muscles. Red and blue arrows symbolize the input signals controlling the pendulum. The red arrow on the screen indicates a perturbation to the right

The EMG controllers put forward in this paper are compared based on a virtual control task of balancing an inverted pendulum in one degree of freedom, as depicted in Fig. 1. In addition to gravity acting on the pendulum, perturbations, in the form of short torque pulses, tip it over and challenge the user to keep it upright. Via EMG measurements of their lower leg muscles, users can modulate a torque acting on the pendulum to steer its movements. To calculate this control torque, four different controllers are used: A *proportional controller*, a *model-based controller* employing a Hill-type muscle model, and two *reflex controllers* (weak and strong) that augment the model-based approach with simulated stretch reflexes. The following sections elaborate on the torque control architectures and conducted user study design.

### EMG-based torque control & virtual control task

#### Hill-type muscle model

The presented torque control architecture contains a Hill-type muscle model, of which two instances are employed for the torque calculation described in Sect. 2.1.3. All parameters and measures listed are general and exist in both instances independently. If a value belongs to muscle 1 or 2 specifically, it is indicated by a 1 or 2 appended to the respective variable’s index.

**Fig. 2:**
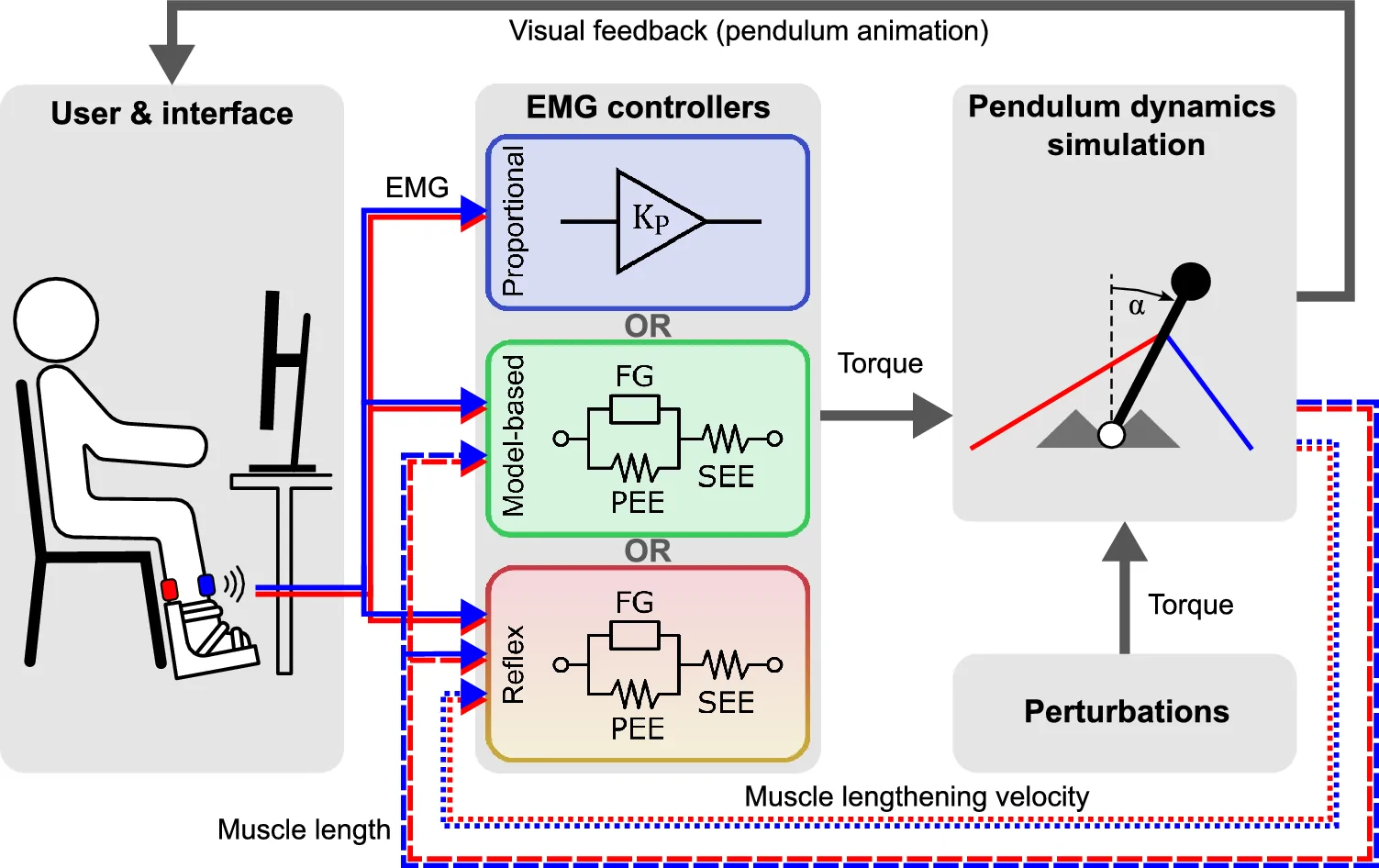
Schematic representation of the experimental setup. Participants wore an orthopedic ankle-foot brace that locks the ankle, while two surface electromyography sensors (marked red and blue) measure activity of lower leg muscles (solid lines). The control torque is calculated by one of three controller types: proportional, model-based and reflex. Together with the perturbations, the control torque influences the pendulum dynamics. Depending on the pendulum position, muscle length is fed back into the model-based and reflex controllers (dashed lines). In addition, muscle lengthening velocity is fed back into the reflex controllers (dotted lines). Through the pendulum display on the screen, visual feedback enables the user to balance the pendulum

Based on the review by Caillet et al.  [31], the model used was designed to consist of components that are well-established in the literature, while keeping the number of parameters low. The latter is meant to ease the individualization of the model to participants in future experiments. The model consists of a force-generating element (FG), parallel elastic element (PEE) and series elastic element (SEE). Its structure is shown in Fig. 2. Given the mechanical arrangement of components, the SEE length $l_\textrm{SEE}$ and FG length $l_\textrm{FG}$ (which is also the PEE length) add up to the total muscle length

1 $$\begin{aligned} l=l_\textrm{SEE}+l_\textrm{FG}, \end{aligned}$$

while there is a force equilibrium

2 $$\begin{aligned} f_\textrm{SEE}=f_\textrm{FG}+f_\textrm{PEE} \end{aligned}$$

between the relative forces of the SEE, FG and PEE in reference to the maximum isometric force $F_0$. The total muscle force *F* is given by

3 $$\begin{aligned} F=F_0 f_\textrm{SEE}. \end{aligned}$$

The FG has a force-length and a force-velocity relationship represented by $f_\textrm{FL}$ and $f_\textrm{FV}$. Its relative force

4 $$\begin{aligned} f_\textrm{FG}=a f_\textrm{FL} f_\textrm{FV} \end{aligned}$$

depends on the activation *a*. The force-length relation is given by

5 $$\begin{aligned} f_\textrm{FL}={\left\{ \begin{array}{ll} 1-\left( \dfrac{l_\textrm{FG}-l_0}{Wl_0}\right) ^2 & ,\ \left|\dfrac{l_{\mathrm{FG}}-l_0}{l_0}\right|\le W \\ 0 & ,\ \text {otherwise} \end{array}\right. }. \end{aligned}$$

The maximum value of one is reached at the optimal FG length $l_0$, decreases quadratically across the width *W*, and becomes zero beyond that  [32, 33].

The force-velocity relation is defined by two rational functions as

6 $$\begin{aligned} f_\textrm{FV}={\left\{ \begin{array}{ll} \dfrac{1+\dfrac{v}{v_\textrm{m}}}{1-\dfrac{v}{a_\textrm{c}v_\textrm{m}}} & ,\ v\le 0\\ f_\textrm{m}-\left( f_\textrm{m}-1\right) \dfrac{1-\dfrac{v}{v_\textrm{m}}}{1+\dfrac{v}{a_\textrm{e} v_\textrm{m}}} & ,\ v> 0 \end{array}\right. }\ , \end{aligned}$$

where *v* is the time derivative of $l_\textrm{FG}$  [34]. For *v=0*, $f_\textrm{FV}$ equals one. It decreases rapidly for concentric contraction (*v≤ 0*), and increases for eccentric contraction (*v>0*) up to the maximum value $f_\textrm{m}$, present at the maximum relaxation speed $v_\textrm{m}$. The velocity *v* is limited to $\pm v_\textrm{m}$. $a_\textrm{c}$ and $a_\textrm{e}$ are shaping parameters for the concentric and eccentric contraction curves, respectively, determining the steepness of $f_\textrm{FV}$ around the static case *v=0*. Finally, the relative forces of the SEE  [35] and PEE  [32] amount to

7 $$\begin{aligned} f_\textrm{SEE}&={\left\{ \begin{array}{ll} k_\textrm{s}\left( \dfrac{l_\textrm{SEE}-l_\textrm{s}}{l_\textrm{s}}\right) ^2 & ,\ l_\textrm{SEE}\ge l_\textrm{s}\\ 0 & ,\ l_\textrm{SEE}<l_\textrm{s} \end{array}\right. } \end{aligned}$$

8 $$\begin{aligned} f_\textrm{PEE}&={\left\{ \begin{array}{ll} \left( \dfrac{l_\textrm{FG}-l_0}{W l_0}\right) ^2 & ,\ l_\textrm{FG}\ge l_0\\ 0 & ,\ l_\textrm{FG}<l_0 \end{array}\right. } \end{aligned}$$

with the SEE slack length $l_\textrm{s}$ and SEE stiffness $k_\textrm{s}$. Equation 8 is synonymous with setting the PEE slack length and stiffness to $l_0$ and $1/W^2$, respectively.

The computation of muscle activation consists of two steps  [36]. Firstly, the delayed input *u'* is calculated by a discrete second-order differential equation

9 $$ \begin{aligned} u^{\prime } (t) = & \left( {1 + p_{a} + p_{b} + p_{a} p_{b} } \right)u(t - T_{d} ) \\ & - \left( {p_{a} + p_{b} } \right)u^{\prime } (t - T_{s} ) - p_{a} p_{b} \;u^{\prime } (t - 2T_{s} ), \\ \end{aligned} $$

with the discrete poles $p_\textrm{a}$ and $p_\textrm{b}$ as well as the delay $T_\textrm{d}$. $T_\textrm{s}$ is the sample time. Secondly, there is the following nonlinear relation between the delayed input and the activation *a* given by

10 $$\begin{aligned} a=\dfrac{\textrm{e}^{Au'}-1}{\textrm{e}^A-1}, \end{aligned}$$

with the shaping parameter *A*.

The geometry of muscle insertion and length dependency was adopted from the modeling of the ankle joint by Geyer and Herr  [37]. The muscle length $l_\textrm{m}$ changes with the joint angle *α * as

11 $$\begin{aligned} l_\textrm{m}=l_0+l_\textrm{s}-r_0\rho \left( \sin \left( \alpha -\alpha _\textrm{m}\right) -\sin \left( \alpha _0-\alpha _\textrm{m}\right) \right) , \end{aligned}$$

where $r_0$ is the maximum lever arm on the joint, $\alpha _\textrm{m}$ is the angle at which the maximum lever arm occurs, and $\alpha _0$ is the angle at which the muscle is just slack with $l_\textrm{m}=l_0+l_\textrm{s}$. The factor *ρ * adjusts the extent of length change and therefore enforces length limits. The lever variable arm *r* of the muscle on the joint is given by

12 $$\begin{aligned} r=r_0\cos \left( \alpha -\alpha _\textrm{m}\right) . \end{aligned}$$

The two muscle model instances used are identical, but oppose each other. The relation of their respective parameters regarding the joint mechanics and insertion come down to

13 $$\begin{aligned} \frac{r_{0,1}}{r_{0,2}}=\frac{\alpha _\textrm{r,1}}{\alpha _\textrm{r,2}} =\frac{\alpha _{0,1}}{\alpha _{0,2}}=-1. \end{aligned}$$

Therefore, if muscle 1 shortens, muscle 2 lengthens and vice versa.

#### Simulated stretch reflex

Reflexes are involuntary responses to various stimuli generated in the spinal cord or brain stem  [20]. They can involve multiple sensory organs and muscles and adapt to different motor tasks. Such complex reflex structures are not applicable to the 1-DoF control task discussed here. Therefore, we focus on emulating the simple stretch reflex – which might be the most important muscle reflex  [20] – to be incorporated in the EMG control architecture. A stretch reflex makes a muscle contract when it gets stretched, while at the same time relaxing its antagonists  [20].

In some existing gait simulation  [37] and prosthesis control  [38] studies, positive force feedback was employed in a simulated reflex scheme. This is a similar approach, but cannot be employed in our control experiment. The mentioned papers switch controllers for different gait phases, interrupting the feedback loop during swing phase. In a continuous control loop like it is put forward in this paper, positive feedback from a muscle’s force to its input signal would create an inherently unstable behavior. Because an increase in muscle activation constitutes a further increase in muscle force, positive force feedback would result in permanent maximum contraction of both antagonistic muscles. Therefore, we base the simulated reflex on the speed of the occurring stretch.

In our model, the reflex signal of a muscle is given by

14 $$\begin{aligned} u_\textrm{r}={\left\{ \begin{array}{ll} K_\textrm{r} \dot{l}_\textrm{m}\dfrac{F-F_\textrm{a}}{F} & ,\ \dot{l}_\textrm{m}>0 \wedge F>F_\textrm{a}\\ 0 & ,\ \text {otherwise} \end{array}\right. }, \end{aligned}$$

where $K_\textrm{r}$ is a constant gain, $\dot{l}_\textrm{m}$ is the time derivative of the muscle length and *F* is the muscle’s force. $F_\textrm{a}$ is the force of the antagonist muscle ($F_\textrm{a,1}=F_2\, ,\ F_\textrm{a,2}=F_1$). When $F_\textrm{a}$ is zero, the reflex is proportional to the muscle lengthening speed with $u_\textrm{r}=K_\textrm{r}\dot{l}_\textrm{m}$ (for $\dot{l}_\textrm{m}>0$). The higher the force of the antagonist is in relation to the respective muscle’s force, the weaker the reflex becomes. This is meant to prevent a reflex to interfere with a quick movement that is driven by the opposing muscle. At all times, the reflex signal $u_\textrm{r}$ is limited to the interval [0, 1].

#### EMG-based torque controllers

In the experiment, three different control architectures are used, one of which comes in two variants, creating a total of four controllers. Firstly, the *model-based controller* calculated the control torque based on the presented hill-type muscle model. Secondly, two *reflex controllers* extend the model-based one by incorporating the reflex model with two different intensities. Lastly, a simple *proportional controller* calculates a control torque without the muscle model.

***Model-based controller***

The model-based controller employs two instances of the hill-type muscle model. They are driven by the EMG input $u_\textrm{EMG}$ as

15 $$\begin{aligned} u=u_\textrm{EMG}. \end{aligned}$$

The control torque *τ * is computed from the forces of the two muscle models as

16 $$\begin{aligned} \tau =F_1 r_1+F_2 r_2. \end{aligned}$$

While all muscle forces are always positive, the muscles act on the pendulum in opposite directions, as depicted in Fig. 2. To represent this geometry, the lever arms have opposite signs in the model (see sect. 2.1.5 for details on model parameters).

***Reflex controllers***

The reflex controllers are based on the same muscle model (including identical parameters), but the EMG amplitude gets combined with the reflex signal to compute the muscle model input *u*, given by

17 $$\begin{aligned} u=u_\textrm{EMG}\left( 1-u_\textrm{r,a}\right) +u_\textrm{r}, \end{aligned}$$

where $u_\textrm{r,a}$ is the antagonist’s reflex. Therefore, a reflex signal increases the same muscle’s input by addition, while impeding the antagonist. This mimics the stretch reflex impacts mentioned in sect. 2.1.2. It is to be noted that because of the opposing orientation of the muscles, $\dot{l}_\textrm{m}$ can only be positive in one muscle at a time. Consequently, there can never be a reflex in both muscles at the same time. Like the reflex signal, the muscle input signal *u* is limited to the interval [0, 1].

To investigate the influence of the reflex strength, a weak and a strong reflex variant are used. Besides the strong reflex controller having an increased gain $K_\textrm{r}$ compared to the weak one, the two are identical.

***Proportional controller***

In the simple proportional controller, the torque was computed directly from the EMG inputs by

18 $$\begin{aligned} \tau =\left( u_\textrm{EMG,2}-u_\textrm{EMG,1}\right) K_\textrm{P} , \end{aligned}$$

with the constant gain $K_\textrm{P}$. The EMG measurements are subtracted from each other, causing the EMG channels to have opposite effects on the control torque.

#### Inverted pendulum dynamics

The virtual inverted pendulum consists of a massless rod with a point mass at its top. It has the angular position *α * and its dynamics are given by

19 $$\begin{aligned} ml^2\ddot{\alpha }=ml\textrm{g}\sin \left( \alpha \right) +\tau +\tau _\textrm{p}, \end{aligned}$$

where *m*, *l*, and $\textrm{g}$ are the mass, pendulum length, and gravitational constant, respectively. The null position (*α =0*) is the upright position, while positive angles represent a tilt to the right. *τ * is the torque exerted by the EMG controller, $\tau _\textrm{p}$ is the perturbation torque introduced in the experimental trials. The perturbations occur as rectangle pulses of $0.5\,\textrm{s}$ duration. All perturbations have the same amplitude ${\hat{\tau }}_\textrm{p}$, but vary in their sign.

The pendulum can only move freely up to $\alpha =\pm 45^\circ$, where it comes to a hard stop. These limits are indicated on the screen by solid gray triangular blocks.

#### Model parameters

The presented equations describing the behavior of the controllers contain a multitude of parameters. Their values shape the model behavior and are therefore an important design element. In general, the pendulum control task is not meant to resemble the behavior of a human leg or body. The focus is rather on providing an intuitive control task with an adequate difficulty that allows to compare the discussed control approaches.

The length of the pendulum is chosen to $l=10\,\textrm{m}$. With the gravitational torque being proportional to *ml* and the inertia being proportional to $ml^2$, the length *l* determines their ratio and therefore how quickly the pendulum tips over. In preliminary tests, these values yielded a manageable pendulum behavior. The pendulum mass was chosen arbitrarily to $m=1\textrm{kg}$. This determines the amount of gravitational torque occurring at every angle. However, how much user input is needed to overcome gravity can be adjusted independently by assigning $F_0$, later on. The gravitational constant is set to $\textrm{g}=9.81\,\mathrm {m/s^2}$.

For the proportional controller, the gain $K_\textrm{P}$ was chosen so that 30 % normalized EMG amplitude is necessary to just overcome gravity at the $\pm 45^\circ$ limit stop. This yields $K_\textrm{P}=23.12\,\textrm{Nm}$.

For the muscle model, the most influential parameters are the maximum isometric force $F_0$ and the length parameters $l_0$ and $l_\textrm{s}$  [31]. Since they determine the relation of joint angle and muscle length, the geometrical parameters $r_0$, *ρ *, $\alpha _\textrm{m}$, and $\alpha _0$ also play a major role in designing the control behavior. The two muscle model instances were decided to be identical. This symmetry does not reproduce the physiological strength relation of plantar- and dorsiflexor muscles, but yields a more intuitive control experiment. The parameters were adopted from the soleus model by Geyer and Herr [37] and are shown in Table 1.

**Table 1:** Muscle model parameters adopted from Geyer and Herr [37]

Parameter	Muscle 1 value	Muscle 2 value
$l_0$	4 cm	4 cm
$l_\textrm{s}$	26 cm	26 cm
$r_0$	−5 cm	5 cm
*ρ *	0.5	0.5
*α*_m_	−20°	20°
*α*_0_	10°	−10°

**Table 2:** Model parameters taken from literature

Parameter	Symbol	Value	Source
Force-length-relation width	*W*	0.56	[32]
Maximum relaxation speed	$v_\textrm{m}$	$12.5 l_0 \dfrac{1}{\textrm{s}}$	[34]
Concentric Force-velocity shape parameter	$a_\textrm{c}$	0.18	[34]
Eccentric Force-velocity shape parameter	$a_\textrm{e}$	$a_\textrm{c}/2=0.09$	[34]
SEE stiffness	$k_\textrm{s}$	625	[35]
EMG input discrete poles	$p_\textrm{a}$, $p_\textrm{b}$	−0.2779, 0.2403	Average from [36]
EMG input delay	$T_\textrm{d}$	40 ms	[36]
EMG input shape parameter	*A*	−1.0105	Average from [36]

By taking these parameters from a gait simulation study  [37], we expect a reasonably natural behavior and resulting length range for the muscle, at least within the range of motion that would be usual for the soleus (interval $[-20^\circ ,40^\circ ]$ for muscle 1, interval $[-40^\circ ,20^\circ ]$ for muscle 2  [37]). To keep the control task challenging and comparable to the proportional controller, it is also important to consider the passive elastic behavior of the muscles. It would be possible to parameterize them in a way so that the nonlinear elastic elements keep the pendulum upright without user input. With the given values for $a_0$, both muscles are slack and therefore forceless for *u=0* within $\pm 10^\circ$. Therefore, no passive assistance is provided in that angular range. To further facilitate an unbiased comparison between the controllers, the maximum isometric force $F_0$ was chosen so that the same input of 30 % is needed to cancel gravity at $\pm 45^\circ$ without changing the length of the FG (*v=0*). The remaining muscle model parameters were taken from literature and are listed in Table 2.

The reflex model only has the single parameter $K_\textrm{r}$ and the influence of its value on the controller performance is part of the scope of this study. At very low values the reflex has negligible influence on pendulum motion, whereas excessively high values over-damp the dynamics, producing unnatural and less intuitive behavior in the control experiment. Some studies suppose a gait-phase dependent stretch reflex contribution of about 50 % to soleus activation  [39]. This relates to the total activation including additional neural signals stimulating the muscle. In the pendulum model, the reflex contribution needs to be defined relative to the maximum input of *u=1*. Since our reflex model also does not incorporate modulation based on gait phase or other system states, a different approach for the parameterization is required. We based $K_\textrm{r}$ around the maximum speed reached by the pendulum tipping over freely. Qualitatively assessing the effects of different gains in preliminary exploratory tests, we chose levels of 5 % and 10 % for $u_\textrm{r}$ for that speed, covering the estimated appropriate range for a noticeable reflex effect without inadequately high damping. This yields $K_\textrm{r}=5.84$ for the weak and $K_\textrm{r}=11.68$ for the strong reflex controller. Because small variations in $K_\textrm{r}$ seemed barely noticeable in the preliminary tests, only these two levels were included in the study.

Finally, the perturbation amplitude was set to ${\hat{\tau }}_\textrm{p}=5.78\,\textrm{Nm}$, which is a fourth of the proportional controller gain $K_\textrm{P}$. Therefore, each perturbation equals an EMG input of $25\,\%$ normalized amplitude for $0.5\,\textrm{s}$. This also was determined in preliminary tests to be a substantial but manageable perturbation strength.

### Experimental setup & user interface

As shown in Fig. 1, users sit in front of a screen that displays the pendulum. They are wearing an orthopedic ankle-foot brace that locks the ankle in a neutral angular position, which serves two purposes. First, it allows users to quickly produce meaningful muscle tensions and EMG amplitudes, because independently of the leg position, the foot can always push against a resistance. Second, it prevents any perception of a connection between the angular positions of the ankle and the pendulum, since the ankle does not move.

EMG activity of their soleus and tibialis anterior muscles is measured as the control input and the following data processing is carried out. The data is measured at 2148 Hz using Delsys Trigno Avanti sensors (Delsys, Natick, MA, USA). The data is first filtered with a second-order $20\,\textrm{Hz}$ Butterworth highpass, then rectified and filtered with a second-order $2\,\textrm{Hz}$ Butterworth lowpass  [10]. The resulting envelope gets normalized by a maximum value determined before the experiment from maximum voluntary contraction. Finally, the resulting EMG envelope is downsampled to the model sample rate of $T_\textrm{s}=500\,\textrm{Hz}$ and fed into the controller as the EMG input signals $u_\textrm{EMG,1}$ (soleus) and $u_\textrm{EMG,2}$ (tibialis anterior).

The calculation of control torque and pendulum dynamics is implemented in Simulink, based on Matlab R2024b (Mathworks, Natick, MA, USA). The model runs at 500 Hz using the Simulink Desktop Real-Time Toolbox.

A two-dimensional animation of the pendulum is displayed in a Matlab App as shown in Fig. 1. Two colored lines (red for muscle 1, blue for muscle 2) illustrate muscles pulling on the pendulum. They increase in width based on the EMG input amplitude, providing feedback about the input to the user. The displayed attachment and corresponding length change of the lines do not match the simulated lever arms and muscle model lengths. The lines rather serve the purpose of confirming an intended control input to the user, helping to move the pendulum in the intended direction. The same is true for the displayed proportions of the pendulum itself. During perturbation occurrences, a red arrow above the pendulum indicates their presence and direction. The desired upright position is illustrated by a dashed silhouette of the upright pendulum.

### Experimental protocol

To compare the controllers, we recruited 16 healthy participants (9 male, 7 female; age: *33.2± 12.1*; body weight: $74.3\pm 14\,\textrm{kg}$) for a repeated-measures design experimental study. All participants gave informed consent. The experimentation was conducted in accordance with the Declaration of Helsinki and a vote from the Ethics Commission at FAU (vote 22-275-S).

The participants were seated in a chair and the EMG electrodes were placed on the soleus and tibialis anterior of their dominant leg (15 right, 1 left). The electrode positions were located by muscle palpation and observation of the EMG signals during flexion and extension of the ankle. The participants then donned the ankle-foot brace. They were free to rest or hold their braced foot in any comfortable position. The participants were introduced to the experimental setup and the control task of balancing the pendulum and keeping it as upright as possible. In a familiarization trial of 1–2 min using the model-based controller, they tried out the setup without perturbations. The model-based controller was used here because in line with the research questions, it acts as a baseline for the performance comparisons in Sect. 3. Afterwards, every participant performed four 2.5-minute trials, one with each controller. The order of controllers presented was randomized to eliminate effects of practice or fatigue on the results. The first 30 s of every trial were not evaluated and contained no perturbations. In the remaining 2 min, 20 perturbations with random signs occurred at randomized times with 3–7 s in between. After each trial, participants reported on the perceived task load by filling out a NASA TLX questionnaire  [40].

#### Evaluation metrics

In accordance with the research questions stated in Sect. 1, the independent variable in our experiment is the choice of EMG controller, and the dependent variables are control performance and user load. To answer the two questions, we mainly consider the comparison of the model-based controller with the proportional controller and the reflex controllers. Differences between the proportional and reflex controllers are not the focus.

To assess control performance, we take the angular root mean square error (RMSE) in relation to the upright position (*α =0*) as the main indicator. Considering an angular range of $\pm 5^\circ$ as successfully balanced, we evaluate the percentage of time spent outside this tolerance as another performance metric. Finally, hitting the $\pm 45^\circ$ limits can be considered a failure in handling the pendulum. Therefore, we also take the number of $\pm 45^\circ$-limit hits as well as the percentage of time in contact with these limits into account.

User load is evaluated based on two metrics. The raw TLX scores  [41] of the NASA TLX questionnaires reflect on the perceived task load of every trial. In addition to this subjective measure, to also consider objective physical effort, we gauge the mean normalized EMG amplitude across both muscles for every trial.

## Results

After accustoming themselves to the system, all participants were able to volitionally control the pendulum movement and lift up the pendulum from the $\pm \,45^\circ$ limits. Despite demonstrating varying skill levels, all participants successfully used all four controllers during the experimental trials.

**Fig. 3:**
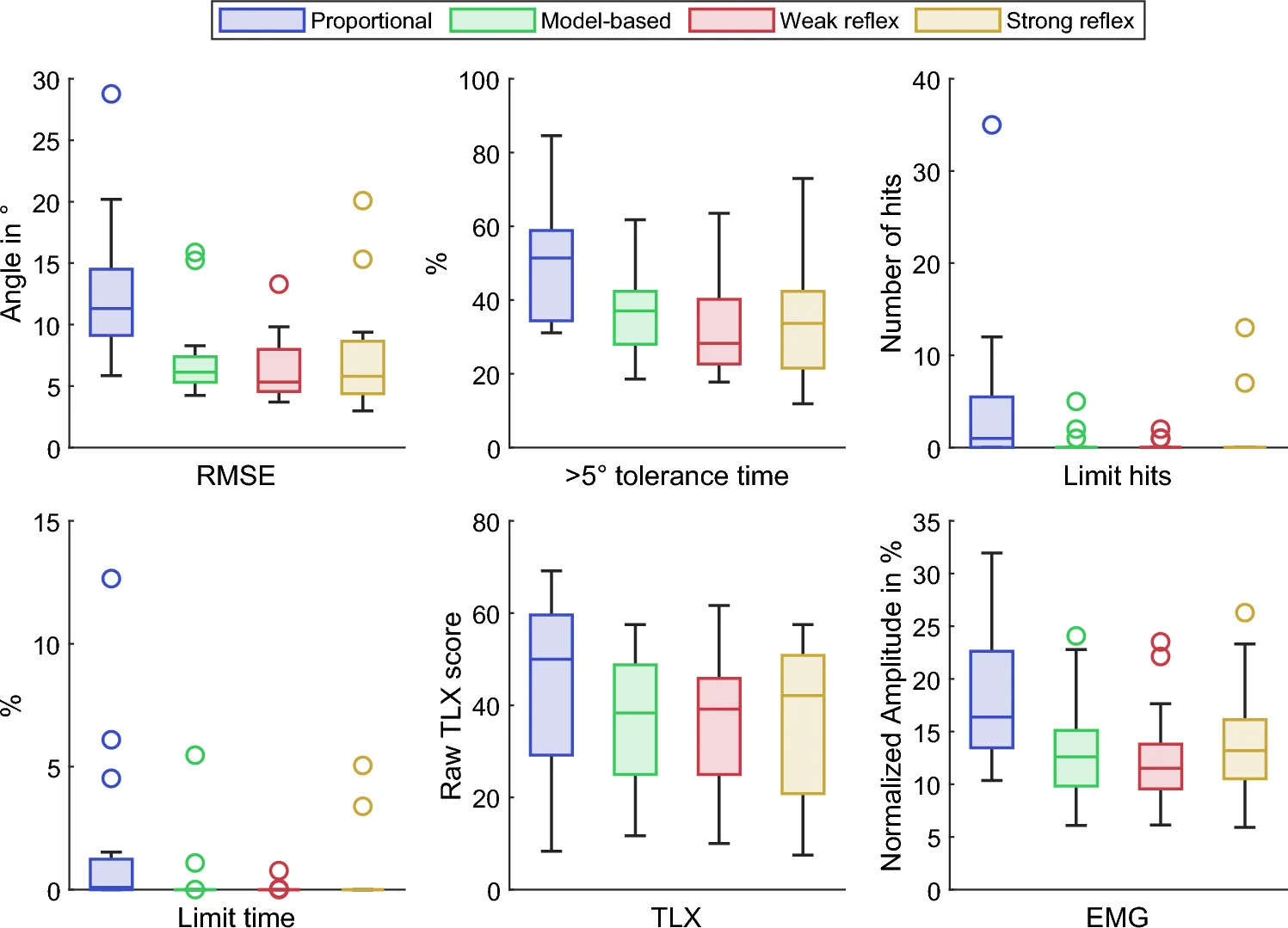
Box plots of the experimental results. The median is denoted by a line in the middle of the box, the upper and lower bounds of the box indicate the 25th and 75th percentile. The whiskers extend to the most extreme data points, except outliers, which are denoted by circles. The proportional controller (blue) shows the least favorable values for all metrics. The weak reflex controller (red) tends to slightly improve upon the model-based controller (green), while the strong reflex controller (yellow) does not

The results for all metrics introduced in Sect. 2.3.1 are presented in Fig. 3 as box plots and summarized in Table 3. For all of the presented metrics, lower values indicate a more successful outcome, i.e., better control performance or lower user load.

To evaluate these results statistically, we performed paired significance tests between the results of the model-based controller and the other three. A Shapiro-Wilk test identified the differences in the number and time of limit hits as well as the EMG amplitude as not normally distributed. We therefore performed a paired t-test on the other three metrics only. The resulting *p*-values were corrected for multiple comparisons using the Holm-Bonferroni method and are presented in Table 4. Since we also cannot assume symmetry in all metrics differences, we used a paired permutation test to incorporate all metrics in a statistical analysis, whose results are presented in Table 5. For the metrics both tests were performed on, the significance results were equal.

**Table 3:**
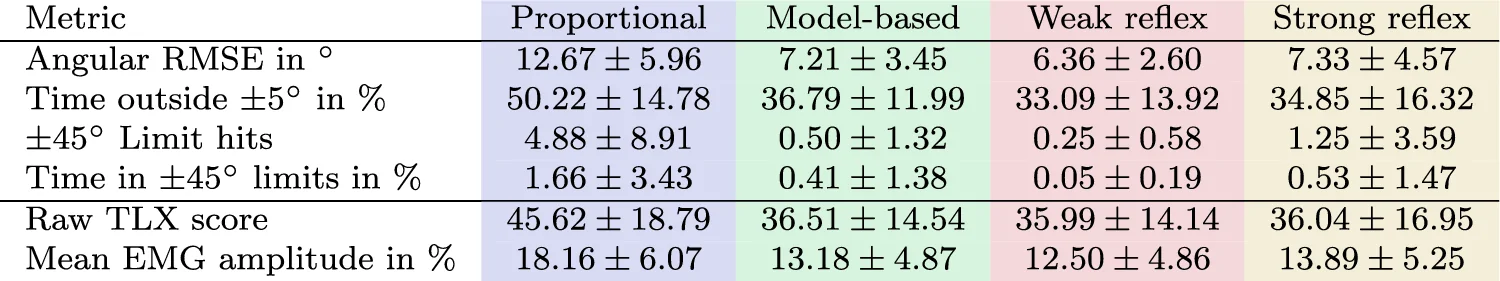
Summary of data results (mean ± standard deviation) for the proportional, model-based, and the weak and strong reflex controllers

**Table 4:**
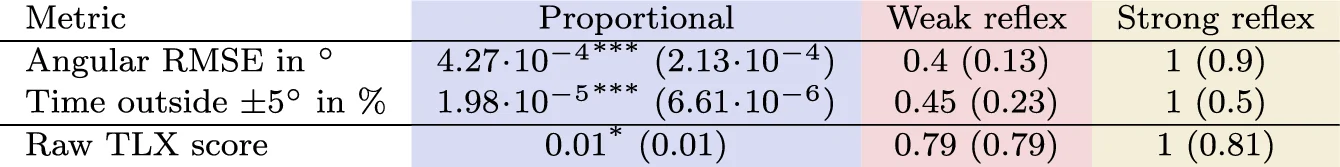
*p*-Values of paired t-tests (Holm-Bonferroni-corrected, raw in brackets) between the model-based controller and the others. Results with statistical significance are marked with one to three asterisks at the 5 %, 1 %, and 0.1 % level, respectively

**Table 5:**
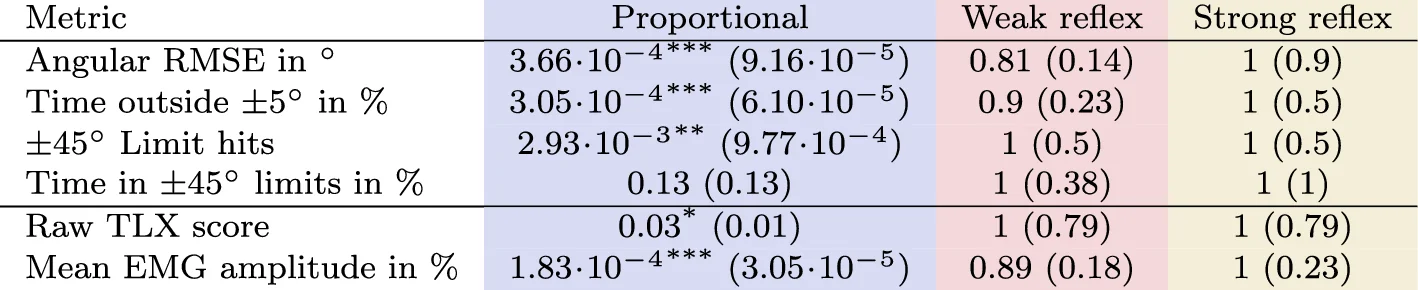
*p*-Values of paired permutation tests (Holm-Bonferroni-corrected, raw in brackets) between the model-based controller and the others. Results with statistical significance are marked with one to three asterisks at the 5 %, 1 %, and 0.1 % level, respectively

As shown in the first subplot of Fig. 3, the proportional controller (blue bar) showed the highest RMSE, with an increase of 76 % compared to the model-based controller (green bar), which was statistically significant. The RMSE did only slightly lower with the weak reflex controller, and slightly increase with the strong one, both without statistical significance. The time outside the $\pm \,5^\circ$ tolerance features very similar results. The proportional controller again had the highest value and a statistically significant increase over the model-based controller. While the weak reflex controller performed best and the strong reflex still slightly improved upon the model-based controller, these differences were also not significant. For both the number and time of the $\pm \,45^\circ$-limit hits, the proportional controller again produced the highest values, while only the difference in the number of hits was significant. Many trials did not feature any limit hits, rendering the comparison of these metrics not very meaningful. Correspondingly, in Fig. 3, the respective box plots for the model-based and reflex controllers lie on the abscissa, with just a few outliers visible. In total, the number of users who hit the limits at all were 11, 3, 3, and 2 for the proportional, model-based controller, weak reflex, and strong reflex controllers, respectively. Therefore, a difference in performance can still be observed between the proportional controller and the others. To summarize the performance metrics, the proportional controller performed lowest in all, with statistical significance in three out of four metrics compared to the model-based controller. The weak reflex controller performed best in all metrics, though only by a small margin and without statistical significance. The strong reflex controller performed slightly inferior compared to the weak one, and occasionally underperformed the model-based controller.

The TLX score and EMG amplitude both differed in a statistically significant manner for the proportional controller compared to the model-based one, with a 25 % and 38 % increase, respectively. The reflex controllers again did not produce significant differences. The TLX score slightly decreased for both, while the EMG amplitude marginally decreased with the weak and increased with the strong reflex.

**Fig. 4:**
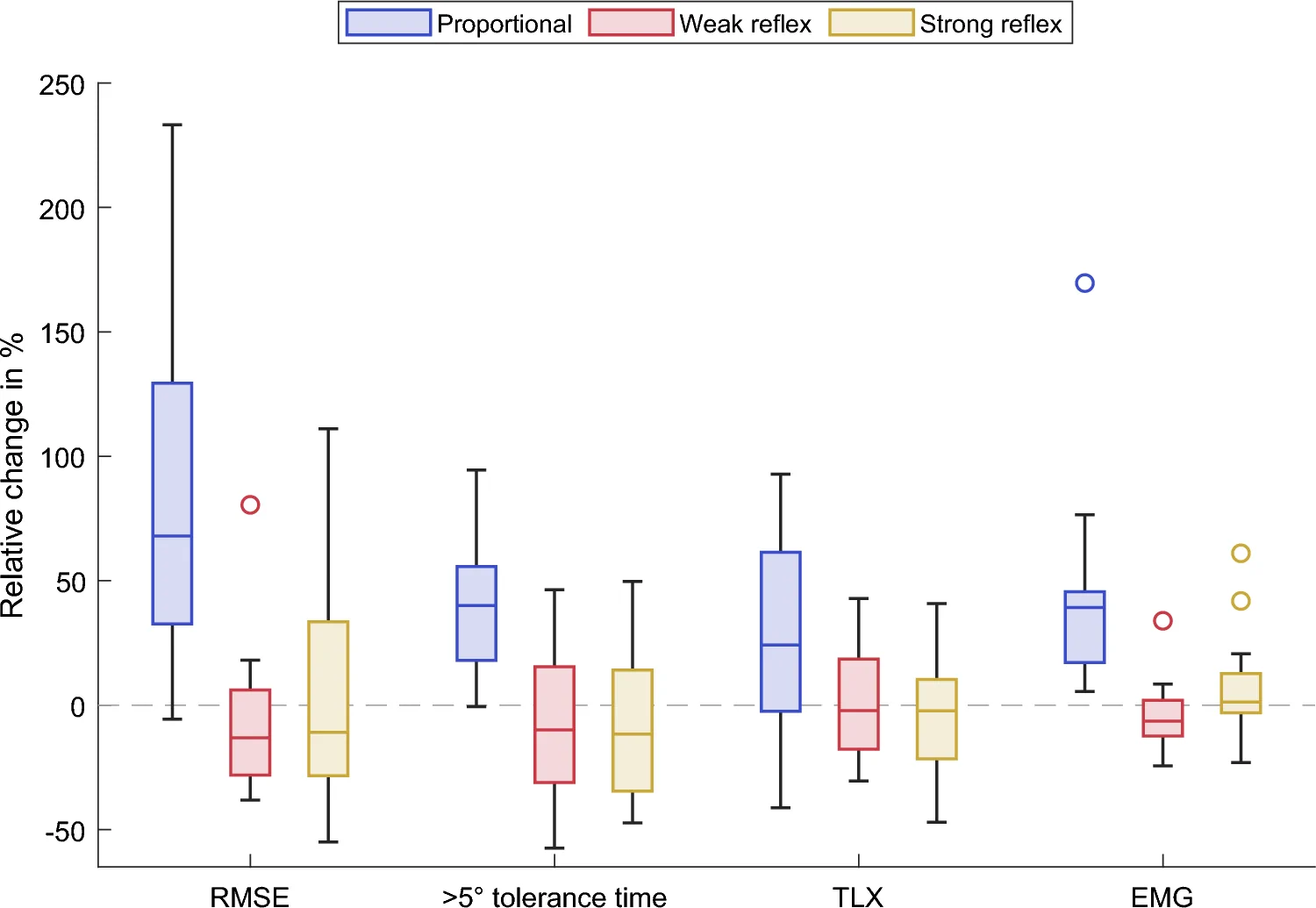
Box plots of the relative performance changes of the proportional and reflex controllers compared to the model-based controller within individual users. The median is denoted by a line in the middle of the box, the upper and lower bounds of the box indicate the 25th and 75th percentile. The whiskers extend to the most extreme data points, except outliers, which are denoted by circles. The dashed line denotes zero, i.e., no change. The proportional controller (blue) strongly exacerbated all metrics. Overall, the reflex controllers (red and yellow) improved the results, to a varying extent and with varying variation

To better comprehend the individual performance changes for each user, we also calculated the relative differences in the introduced metrics within the trials of individual participants. Figure 4 and Table 6 show the average percentage changes in results (again, lower is better) that the users experienced for the reflex and proportional controllers in relation to the model-based controller as a base frame. Note that since the optimal value for all metrics is zero, the maximum possible improvement is a reduction by $-100\,\%$, while the relative increase can be infinite. The limit hits and time in limits were not included, since many trials contained no limit hits at all, which causes a division by zero when calculating the relative change.

**Table 6:**
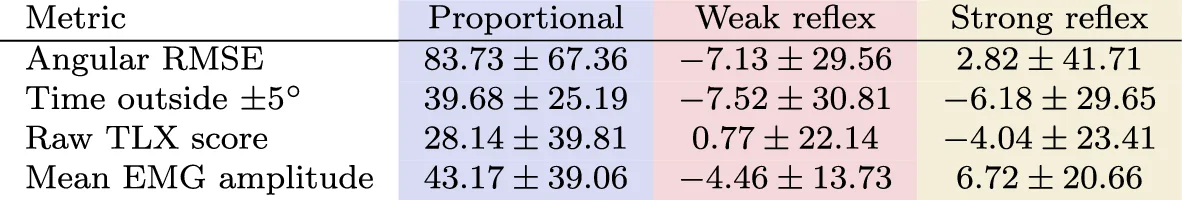
Relative performance changes (mean ± standard deviation) of the reflex and proportional controllers compared to the model-based controller

The RMSE and time outside the tolerance greatly exacerbated for almost all participants with the proportional controller. This can be observed in Fig. 4, as the whiskers of the first two blue boxes hardly extend to below the dashed zero line. The reflex controllers (red and yellow) improved these control performance metrics for most users, but not for all. Here, the medians all show at least a $-10\,\%$-improvement, while in the mean, the RMSE was higher for the strong reflex. The raw TLX score deteriorated by $28\,\%$ for the proportional controller. It did not differ notably for the reflex controllers, with their median almost lying at the zero line. The EMG amplitude increased with the proportional controller for all users. The weak reflex controller slightly decreased it, with the strong reflex controller slightly increasing it.

## Discussion

In this paper, we present a muscle-model-based EMG torque controller and extend it by the novel approach of including simulated muscle reflexes. In a repeated-measures design user study, these controller variants are compared to each other and to a simple proportional EMG controller in a quick to set up and intuitive to use virtual control experiment. The differences in trial results demonstrate the relevance of controller formulation and reassure the necessity to further engage in this development challenge  [3].

Between the model-based and proportional controllers, a clear distinction in performance can be observed, as the proportional controller showed less desirable results in all metrics considered. For five out of six metrics, these improvements were statistically significant. Regarding the first research question, the results let us conclude that the model-based controller strongly improves control performance and user load upon the proportional one. A feasible explanation for why the proportional controller was the most challenging lies in the angle-torque behavior the controllers provide. The proportional controller provides the same torque leverage on the pendulum for the full range of motion. However, the load exerted by gravity is sinusoidal and increases with the joint angle *α *. Therefore, high EMG amplitudes are required at large angles, while very precise inputs are needed for fine control around the upright position. In contrast, the torque achievable with the model-based control varies with *α * and its derivative, inherently stabilizing the pendulum. At $|\alpha |>10\,^\circ$, the muscle responsible for lifting the pendulum back up is stretched. The PEE helps to lift the pendulum without user input, and the force at a given input amplitude is increased. Therefore, the descent of user muscle contraction that is asked for is not as steep between big and small joint angles. At the same time, the antagonist (which would pull the pendulum down further) is shortened below its optimal length, providing a decreased force output. Involuntary co-contractions therefore also hinder the uplift of the pendulum less than with the proportional controller. The force-velocity relation further assists the deceleration of the pendulum, since the elongating muscle increases in strength.

These results support the assumption that incorporating muscle mechanics improves controllability in EMG-based prosthesis interfaces. We suppose the stabilizing tendencies of the model-based controller will benefit a real prosthesis use with different model parameters in a similar way. This supports the use of such model-based approaches in recent weight-bearing prosthesis experiments  [21, 22, 42]. Also, co-contraction is a known issue in EMG control for people with a transtibial amputation  [12], whose effect is also reduced here.

The second research question concerns the same performance and user load distinctions, but regarding the incorporation of simulated reflexes. For the presented reflex controllers, there was no statistically significant improvement in results when comparing them with the model-based EMG controller. Yet, the boxplots in Fig. 4 do show some tendencies. While the TLX score barely differs compared to the model-based controller, indicating no change in the perceived task load by the users, all other metrics show a tendency towards improvement, particularly for the weak reflex controller with a lower reflex gain. For at least half of the participants, the RMSE, time outside $\pm 5^\circ$ and the mean EMG amplitude were reduced by at least $13\,\%$, $10\,\%$ and $6\,\%$, respectively. The strong reflex controller does not further improve these results with its twice as strong reflex. For the mean EMG amplitude, the median change is even positive. While we see an indication that the reflex model does change the control behavior, showing improvements performance and user load, these results do not allow us to confirm a generally improved control behavior with certainty.

As an explanation for the minor performance impact, one could assume that the reflex gain $K_\textrm{r}$ was too low to produce meaningful muscle activations. However, between participants, the average maximum amplitude of the reflex signal $u_\textrm{R}$ was $5.53\,\%$ for the weak and $12.47\,\%$ for the strong reflex controller. Given that only $30\,\%$ input is needed to compensate for gravity at the maximum joint angles, at least the reflex of the strong reflex controller must have had a non-negligible effect. We assume that the reflex model indeed helped to stop the pendulum from tipping over, but also damped the momentum during raising. This might have made the system less intuitive to use, especially for the stronger variant, like it was observed in the exploratory tests for high reflex gains. While an undetected optimal intermediate value for $K_\textrm{r}$ could exist, the overall similarity of the two reflex controller’s results let us doubt significant performance improvements from it.

We suspect the cause of an absence of significant improvements in the reflex controllers to lay in the simplified stretch-only response. Biologically, reflex gains change with task state, load, contraction velocity, and antagonist coactivation [20, 43]. A possible remedy for this, in future work, could be the incorporation of muscle force into the current model, either by combining velocity and force feedback, or by force-dependent gating of the reflex. This could reduce reflex effects on high-momentum movements at low muscle forces. Alternatively, more complex reflex models with a biomimicry structure could be explored, like described by Graham and Redman  [43]. Luo et al.  [24] accomplished noticeable improvements in both volitional force control and multiple functional tasks by incorporating a biomimicry reflex model in a hand prosthesis.

A possible change in the experimental setup would be the removal of the arrows indicating perturbations. While this might make the pendulum’s behavior less comprehensible overall, it would likely slow down user reactions, better highlighting the reflexes’ effects on unexpected movements.

All participants familiarized themselves with the setup and control task using the model-based controller, which could have created a bias towards it. However, participants did not practice the handling of perturbations before the actual trials, which was a main challenge of the task with a major impact on the results. Additionally, every trial included a 30 s familiarization period before the evaluated time frame, which gave the users considerable time to get used to the new controller in comparison to the time they spent using the model-based controller beforehand. Since every experimental trial exceeds the initial familiarization in length, we suspect a possible learning bias to only be relevant for participants who started with the model-based controller, and do not suppose a notable impact on the overall results. To further reduce possible bias effects, future studies could carry out a balanced familiarization across all controller variants.

Overall, our experimental setup does not replicate the use of an ankle-foot prosthesis, though some components and parameters of our torque control scheme based on two Hill-type muscle models are inspired by human kinematics. The visual feedback regarding the control task is unnatural, while other sensory inputs like proprioception in adjacent body parts, equilibrioception and sense of load on the limb are all missing or irrelevant. Additionally, the symmetry of antagonist muscles in the setup was sensible for this experiment, but does not reflect the behavior and strength of human lower-limb muscles. A similar control task with prosthesis hardware, e.g., holding balance in the sagittal plane while standing, would differ in dynamic characteristics, especially the inertia and the controllable range of motion. Therefore, future work should incorporate realistic prosthesis experiments, which might yield different results for the same selection of controllers. Successive studies could also look for deeper insights into user experience, incorporating other questionnaires like the System Usability Scale  [44], the User Experience Questionnaire  [45], or anecdotal feedback to explore unforeseen usability aspects.

## Conclusion

In this study, we empirically demonstrated the benefits of a model-based EMG controller in a virtual EMG control task, compared to proportional control. With this rare direct comparison in a repeated-measures design, we help closing the gap of EMG control method comparative analysis, confirming the suitability of the model-based approach. Additionally, we presented a novel combination of volitional EMG control with simulated reflexes. Besides control performance, we also observed user load, gaining a more comprehensive insight. While the reflex model could not significantly improve the control outcomes, the results suggest that with further adjustments, it could benefit users in similar or more complex EMG control tasks.

In the future, we will take the comparison of (model-based) EMG controllers to prosthesis hardware and test standing balance in the sagittal plane, before successively extending this approach to walking. The virtual experimental setup described in this paper can further be used to conduct preliminary tests on new reflex model designs at little expense. Finally, we expect that the control methods and findings outlined in this paper will serve to improve usability and performance in assistive robotic devices.

## References

[1] Berry D. Microprocessor Prosthetic Knees. Phys Med Rehabil Clin N Am. 2006;17(1):91–113. 10.1016/j.pmr.2005.10.006. 16517347

[2] Burçak B, Kesikburun B, Köseoğlu BF, Öken Ö, Doğan A. Quality of life, body image, and mobility in lower-limb amputees using high-tech prostheses: A pragmatic trial. Ann Phys Rehabil Med. 2021;64(1):101405. 10.1016/j.rehab.2020.03.016. 32561506

[3] Voloshina AS, Collins SH. Lower limb active prosthetic systems—overview. In: Wearable Robotics, pp. 469–486. Academic Press, San Diego (2020). 10.1016/B978-0-12-814659-0.00023-0.

[4] Bekrater-Bodmann R. Factors Associated With Prosthesis Embodiment and Its Importance for Prosthetic Satisfaction in Lower Limb Amputees. Front Neurorobot. 2021;14:10.3389/fnbot.2020.604376 PMC7843383 33519413

[5] Manz S, Valette R, Damonte F, Avanci Gaudio L, Gonzalez-Vargas J, Sartori M, et al. A review of user needs to drive the development of lower limb prostheses. J Neuroeng Rehabil. 2022;19(1):119. 10.1186/s12984-022-01097-1. PMC9636812 36335345

[6] Christ O, Beckerle P, Rinderknecht S, Vogt J. Usability, satisfaction and appearance while using lower limb prostheses: Implications for the future. Neurosci Lett. 2011;500:50. 10.1016/j.neulet.2011.05.214.

[7] Baars EC, Schrier E, Dijkstra PU, Geertzen JHB. Prosthesis satisfaction in lower limb amputees: A systematic review of associated factors and questionnaires. Medicine. 2018;97(39):12296. 10.1097/MD.0000000000012296. PMC6181602 30278503

[8] Zheng X. Neural Interface: Frontiers and Applications. In: Advances in Experimental Medicine and Biology. Singapore: Springer; 2019. 10.1007/978-981-13-2050-7_7 31729676

[9] Huang S, Huang H. Voluntary Control of Residual Antagonistic Muscles in Transtibial Amputees: Feedforward Ballistic Contractions and Implications for Direct Neural Control of Powered Lower Limb Prostheses. IEEE Trans Neural Syst Rehabil Eng. 2018;26(4):894–903. 10.1109/TNSRE.2018.2811544. 29641394

[10] Huang S, Huang H. Voluntary Control of Residual Antagonistic Muscles in Transtibial Amputees: Reciprocal Activation, Coactivation, and Implications for Direct Neural Control of Powered Lower Limb Prostheses. IEEE Trans Neural Syst Rehabil Eng. 2019;27(1):85–95. 10.1109/TNSRE.2018.2885641. 30530332

[11] Yadav D, Veer K. Recent trends and challenges of surface electromyography in prosthetic applications. Biomed Eng Lett. 2023. 10.1007/s13534-023-00281-z. PMC10382439 37519867

[12] Huang S, Ferris DP. Muscle activation patterns during walking from transtibial amputees recorded within the residual limb-prosthetic interface. J Neuroeng Rehabil. 2012;9(1):55. 10.1186/1743-0003-9-55. PMC3582563 22882763

[13] Alcaide-Aguirre RE, Morgenroth DC, Ferris DP. Motor control and learning with lower-limb myoelectric control in amputees. J Rehabilit Res Develop. 2013;50(5):687. 10.1682/JRRD.2012.06.0115. 24013916

[14] Fleming A, Huang S, Huang H. Coordination of Voluntary Residual Muscle Contractions in Transtibial Amputees: A Pilot Study. In: 2018 40th Annual International Conference of the IEEE Engineering in Medicine and Biology Society (EMBC), pp. 2128–2131 (2018). 10.1109/EMBC.2018.8512674 30440824

[15] Fleming A, Stafford N, Huang S, Hu X, Ferris DP, Huang HH. Myoelectric control of robotic lower limb prostheses: A review of electromyography interfaces, control paradigms, challenges and future directions. J Neural Eng. 2021;18(4):041004. 10.1088/1741-2552/ac1176. PMC8694273 34229307

[16] Ahkami B, Ahmed K, Thesleff A, Hargrove L, Ortiz-Catalan M. Electromyography-based control of lower limb prostheses: A systematic review. IEEE Transactions on Medical Robotics and Bionics, 1–1 (2023) 10.1109/TMRB.2023.3282325 PMC10470657 37655190

[17] Cimolato A, Driessen JJM, Mattos LS, De Momi E, Laffranchi M, De Michieli L. EMG-driven control in lower limb prostheses: A topic-based systematic review. J Neuroeng Rehabil. 2022;19(1):43. 10.1186/s12984-022-01019-1. PMC9077893 35526003

[18] Gehlhar R, Tucker M, Young AJ, Ames AD. A review of current state-of-the-art control methods for lower-limb powered prostheses. Annu Rev Control. 2023;55:142–64. 10.1016/j.arcontrol.2023.03.003 PMC10449377 37635763

[19] Voß M, Koelewijn AD, Beckerle P. Intuitive and versatile bionic legs: A perspective on volitional control. Front Neurorobot. 2024;18:1410760. 10.3389/fnbot.2024.1410760. PMC11225306 38974662

[20] Kandel ER, Schwartz JH, Jessell TM, Siegelbaum SA, Hudspeth AJ. Principles of Neural Science. 5th ed. New York: McGraw-Hill Education Ltd; 2012.

[21] Shu T, Shallal C, Chun E, Shah A, Bu A, Levine D, et al. Modulation of prosthetic ankle plantarflexion through direct myoelectric control of a subject-optimized neuromuscular model. IEEE Robot Auto Lett. 2022;7(3):7620–7. 10.1109/LRA.2022.3183762.

[22] Abdelbar M, Örn Sverrisson A, Briem K, Lecomte C, Brynjólfsson S. A Hybrid Controller Integrating Finite-State Impedance and Electromyography-Driven Musculoskeletal Model for Robotic Active Ankle Prostheses: A Case Study. IEEE Access. 2024;12:157329–45. 10.1109/ACCESS.2024.3485570.

[23] Niu CM, Luo Q, Chou C-H, Liu J, Hao M, Lan N. Neuromorphic model of reflex for realtime human-like compliant control of prosthetic hand. Ann Biomed Eng. 2021;49(2):673–88. 10.1007/s10439-020-02596-9. PMC7851042 32816166

[24] Luo Q, Chou C-H, Liang W, Tang H, Du R, Wei K, et al. Reflex regulation of model-based biomimetic control for a tendon-driven prosthetic hand. Biomed Signal Process Control. 2025;101:107223. 10.1016/j.bspc.2024.107223.

[25] Hill AV (1938) The heat of shortening and the dynamic constants of muscle. In: Proceedings of the Royal Society of London. Series B-Biological Sciences 126(843), 136–195

[26] Wang J, Kannape OA, Herr HM. Proportional EMG control of ankle plantar flexion in a powered transtibial prosthesis. In: 2013 IEEE 13th International Conference on Rehabilitation Robotics (ICORR), pp. 1–5. IEEE, Seattle, WA (2013). 10.1109/ICORR.2013.6650391 24187210

[27] Hunt GR, Hood S, Lenzi T. Stand-Up, Squat, Lunge, and Walk With a Robotic Knee and Ankle Prosthesis Under Shared Neural Control. IEEE Open J Eng Med Biol. 2021;2:267–77. 10.1109/OJEMB.2021.3104261 PMC8901006 35402979

[28] Fleming A, Liu W, Huang HH. Neural prosthesis control restores near-normative neuromechanics in standing postural control. Sci Robot. 2023;8(83):5758. 10.1126/scirobotics.adf5758. PMC10882517 37851818

[29] Fleming A, Huang S, Huang H. Proportional Myoelectric Control of a Virtual Inverted Pendulum Using Residual Antagonistic Muscles: Toward Voluntary Postural Control. IEEE Trans Neural Syst Rehabil Eng. 2019;27(7):1473–82. 10.1109/TNSRE.2019.2922102. 31180864

[30] Voß M, Beckerle P. Integrating simulated muscle reflexes with volitional inputs to enhance lower-limb EMG-control. In: 12th International Symposium on Adaptive Motion of Animals and Machines (AMAM 2025). Darmstadt, Germany; 2025. 10.26083/tuprints-00030960.

[31] Caillet AH, Phillips ATM, Carty C, Farina D, Modenese L. Hill-Type Models of Skeletal Muscle and Neuromuscular Actuators: A Systematic Review. IEEE Rev Biomed Eng. 2026;19:159–81. 10.1109/RBME.2025.3593185. 40938713

[32] Van Soest AJ, Bobbert MF. The contribution of muscle properties in the control of explosive movements. Biol Cybern. 1993;69(3):195–204. 10.1007/BF00198959. 8373890

[33] Gallucci JG, Challis JH. Examining the Role of the Gastrocnemius during the Leg Curl Exercise. J Appl Biomech. 2002;18(1):15–27. 10.1123/jab.18.1.15.

[34] Van Ruijven LJ, Weijs WA. A new model for calculating muscle forces from electromyograms. Eur J Appl Physiol. 1990;61(5–6):479–85. 10.1007/BF00236071. 2079070

[35] Bobbert MF, Van Ingen Schenau GJ. Isokinetic plantar flexion: Experimental results and model calculations. J Biomech. 1990;23(2):105–19. 10.1016/0021-9290(90)90345-4. 2312517

[36] Lloyd DG, Besier TF. An EMG-driven musculoskeletal model to estimate muscle forces and knee joint moments in vivo. J Biomech. 2003;36(6):765–76. 10.1016/S0021-9290(03)00010-1. 12742444

[37] Geyer H, Herr H. A Muscle-Reflex Model That Encodes Principles of Legged Mechanics Produces Human Walking Dynamics and Muscle Activities. IEEE Trans Neural Syst Rehabil Eng. 2010;18(3):263–73. 10.1109/TNSRE.2010.2047592. 20378480

[38] Thatte N, Duan H, Geyer H. A Method for Online Optimization of Lower Limb Assistive Devices with High Dimensional Parameter Spaces. In: 2018 IEEE International Conference on Robotics and Automation (ICRA), pp. 5380–5385 (2018). 10.1109/ICRA.2018.8460953

[39] Zehr EP, Stein RB. What functions do reflexes serve during human locomotion? Prog Neurobiol. 1999;58(2):185–205. 10.1016/S0301-0082(98)00081-1. 10338359

[40] Hart SG, Staveland LE. Development of NASA-TLX (Task Load Index): Results of Empirical and Theoretical Research. In: Hancock, P.A., Meshkati, N. (eds.) Advances in Psychology. Human Mental Workload, vol. 52, pp. 139–183. North-Holland, Amsterdam (1988). 10.1016/S0166-4115(08)62386-9

[41] Hart SG. Nasa-Task Load Index (NASA-TLX); 20 Years Later. Proceed Human Factor Ergonomic Soc Ann Meeting. 2006;50(9):904–8. 10.1177/154193120605000909.

[42] Shah C, Fleming A, Nalam V, Liu M, Huang HH. Design of EMG-driven Musculoskeletal Model for Volitional Control of a Robotic Ankle Prosthesis. In: 2022 IEEE/RSJ International Conference on Intelligent Robots and Systems (IROS), pp. 12261–12266. IEEE, Kyoto, Japan (2022). 10.1109/IROS47612.2022.9981305

[43] Graham BP, Redman SJ. Dynamic behaviour of a model of the muscle stretch reflex. Neural Netw. 1993;6(7):947–62. 10.1016/S0893-6080(09)80005-1.

[44] Brooke J. SUS - A quick and dirty usability scale. Usability Eval Indust. 1996;189(194):4–7.

[45] Laugwitz B, Held T, Schrepp M. Construction and Evaluation of a User Experience Questionnaire. In: Holzinger, A. (ed.) HCI and Usability for Education and Work vol. 5298, pp. 63–76. Springer, Berlin, Heidelberg (2008). 10.1007/978-3-540-89350-9_6

